# Circulating eosinophils associated with responsiveness to COVID-19 vaccine and the disease severity in patients with SARS-CoV-2 omicron variant infection

**DOI:** 10.1186/s12890-023-02473-w

**Published:** 2023-05-22

**Authors:** Zhuxian Zhu, Jixu Cai, Qiang Tang, Yin-yuan Mo, Tiantian Deng, Xiaoyu Zhang, Ke Xu, Beishou Wu, Haicheng Tang, Ziqiang Zhang

**Affiliations:** 1 Department of Nephrology, Tongji Hospital, Tongji University School of Medicine, Shanghai, China; 2 Department of Emergency Medicine, Tongji University School of Medicine, Shanghai, China; 3 Department of Emergency, Shanghai Public Health Clinical Center, Fudan University, Shanghai, China; 4 Institute of Clinical Medicine, Zhejiang Provincial People’s Hospital of Hangzhou Medical College, Hangzhou, China; 5 Shanghai Nanxiang Community Health Service Center, Shanghai, China; 6 Section of Education, Shanghai Public Health Clinical Center, Fudan University, Shanghai, China; 7 Department of General Medicine, Tongji University School of Medicine, Shanghai, China; 8 Department of Respiratory Medicine, Shanghai Public Health Clinical Center, Fudan University, 2901 Caolang Road, Shanghai, 201508 China; 9 Department of Infectious Disease & Department of Respiratory and Critical Care Medicine, Tongji Hospital, Tongji University School of Medicine, 389 Xincun Road, Shanghai, 200065 China

**Keywords:** COVID-19 vaccine, SARS-CoV-2, Omicron variant, Eosinophil (EOS), T cell immunity, Disease severity

## Abstract

**Objective:**

This study aimed to investigate the longitudinal circulating eosinophil (EOS) data impacted by the COVID-19 vaccine, the predictive role of circulating EOS in the disease severity, and its association with T cell immunity in patients with SARS-CoV-2 Omicron BA.2 variant infection in Shanghai, China.

**Methods:**

We collected a cohort of 1,157 patients infected with SARS-CoV-2 Omicron/BA.2 variant in Shanghai, China. These patients were diagnosed or admitted between Feb 20, 2022, and May 10, 2022, and were classified as asymptomatic (n = 705), mild (n = 286) and severe (n = 166) groups. We compiled and analyzed data of patients’ clinical demographic characteristics, laboratory findings, and clinical outcomes.

**Results:**

COVID-19 vaccine reduced the incidence of severe cases. Severe patients were shown to have declined peripheral blood EOS. Both the 2 doses and 3 doses of inactivated COVID-19 vaccines promoted the circulating EOS levels. In particular, the 3rd booster shot of inactivated COVID-19 vaccine was shown to have a sustained promoting effect on circulating EOS. Univariate analysis showed that there was a significant difference in age, underlying comorbidities, EOS, lymphocytes, CRP, CD4, and CD8 T cell counts between the mild and the severe patients. Multivariate logistic regression analysis and ROC curve analysis indicate that circulating EOS (AUC = 0.828, p = 0.025), the combination of EOS and CD4 T cell (AUC = 0.920, p = 0.017) can predict the risk of disease severity in patients with SARS-CoV-2 Omicron BA.2 variant infection.

**Conclusions:**

COVID-19 vaccine promotes circulating EOS and reduces the risk of severe illness, and particularly the 3rd booster dose of COVID-19 vaccine sustainedly promotes EOS. Circulating EOS, along with T cell immunity, may have a predictive value for the disease severity in SARS-CoV-2 Omicron infected patients.

## Background

COVID-19 epidemic remains to be a matter of great concern internationally [1–6]. Since the World Health Organization (WHO) listed the COVID-19 Omicron mutant as a noteworthy mutant in November 2021 [7], SARS-CoV-2 (severe acute respiratory syndrome coronavirus 2) Omicron/BA.2 variant has quickly replaced the Delta variant and become the main epidemic strain. Considering the rapid transmission, the widespread SARS-CoV-2 Omicron variant infection has been a public health emergency of international concern [8–10]. As of May 10, 2022, more than 600,000 cases with SARS-CoV-2 Omicron BA.2. variant infection had been documented in Shanghai, China (http://sh.bendibao.com).

Eosinopenia has been linked to COVID-19 [11–13], however, the sample size in these studies was limited, and the detailed role of eosinophils (EOS) in SARS-CoV-2 Omicron variant infected patients remains unclear. A study has indicated that eosinophilia is associated with improved COVID − 19 outcomes in patients treated with inhaled corticosteroid [14]. A study has introduced a risk stratification score that includes eosinophils at < 5 per microliter to identify patients who are likely to be manifesting with COVID-19. However, the mechanism by which eosinophilia associated with COVID-19 remains unclear. Moreover, little is known about the role of inactivated COVID-19 vaccine on peripheral blood EOS. In this study, we investigated the severity predictive role of circulating EOS and its association with T cell immunity, in patients with SARS-CoV-2 Omicron BA.2 variant infection in Shanghai, China.

Compared with the original virus strain, Omicron variants have had remarkable shifts and evolution [15, 16]. Recent studies have indicated that SARS-COV-2 Omicron variants have shown immune escape to the existing COVID-19 vaccine [17, 18]. In this study, we found that inactivated COVID-19 vaccine could reduce the incidence of severe cases. We also evaluated the longitudinal EOS data impacted by the COVID-19 vaccine. Interestingly, we found inactivated COVID-19 vaccine sustainedly promoted circulating EOS. Thus, in addition to the specific immunity of the COVID-19 vaccine against SARS-CoV-2, we tried to clarify the potential role of EOS associated with COVID-19 vaccine-mediated antiviral response.

## Patients and methods

### Data sources

We conducted a multicenter, cross-sectional study. Data were collected from the laboratory-confirmed SARS-CoV-2 infected patients from the recent COVID-19 pandemic of 2022 in Shanghai, China. Patients involved in this study were diagnosed or admitted to Tongji Hospital and Shanghai Public Health Clinical Center in Shanghai from Feb 20, 2022, through May 10, 2020. Patients younger than 18 years old, and other patients who did not meet the criteria were excluded from this study. Inactivated COVID-19 vaccines were from Sinovac Life Sciences Co., Ltd. (China). This retrospective study was approved by the Ethics Committee of Tongji Hospital (No. K-KYSB-2020-189) and granted a waiver of informed consent from study participants.

**Fig. 1:**
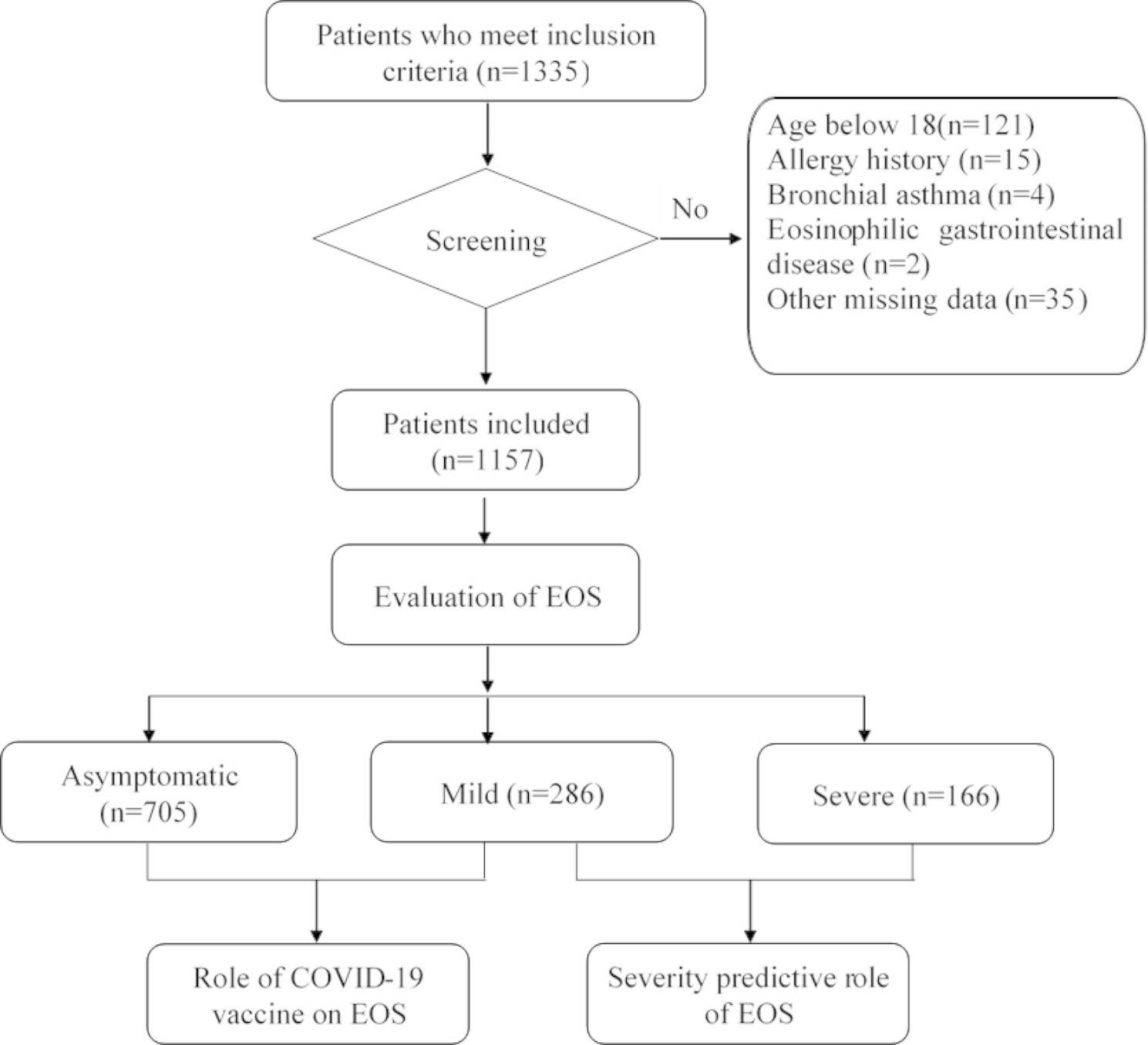
Schematic of study design EOS, Eosinophils; COVID-19, 2019 coronavirus disease

A total of 1335 patients with confirmed SARS-CoV-2 Omicron BA.2 variant infection were considered in this study. Patients were excluded if they had an age below 18 years (n = 121), eosinophilia(n = 1), allergy history (n = 15), bronchial asthma (n = 4) or missed other relevant data (n = 35). Eventually, a total of 1157 patients were involved and divided into asymptomatic group (n = 705), mild group (n = 286), and severe groups (n = 166). The severe group includes the severe patients and critical severe patients, according to disease severity defined by the WHO guidance [19]. Severe patients with intensive care unit admission required high-flow nasal oxygen (HFNO), an intubation followed by mechanical ventilation. 69 out of 166 severe patients had a COVID-19-related death. The schematic diagram of this study was shown in Fig. 1.

### Data collection

Data of the medical history and laboratory findings were collected from electronic medical records. Laboratory tests include white blood cell (WBC) counts, counts of neutrophils, lymphocytes, monocytes, Eosinophils (EOS), and C-reactive protein (CRP), D-dimer、CD4、CD8、CD4/CD8、NLR(Neutrophil-to-Lymphocyte Ratio). Medical history include Vaccination history、hypertension、coronary artery disease、diabetes、COPD、cerebrovascular disease、chronic renal disease、malignant tumor、Rheumatic disease.

### Definitions

The current round of COVID-19 epidemic in Shanghai has been confirmed as SARS-CoV-2 Omicron/BA.2 variant infection by the Shanghai Municipal Center for Disease Control and Prevention. The disease severity was defined according to the latest WHO guidance [19]. Severe patients were admitted to the intensive care unit. Decreased circulating EOS was defined as the counts of peripheral blood EOS under the lower limit of the normal range (< 0.02 × 10^9^/L).

### Statistical analysis

Statistical analysis was performed by SPSS (NY, USA). Data ware shown as medians with interquartile ranges (IQR) for continuous variables and numbers with percentages for categorical variables. The Mann-Whitney U test is used to compare continuous variables between the asymptomatic and the mild, or the mild and severe groups. The chi-square test or Fisher’s exact test was used to compare the categorical variables. Significant independent variables in univariate analysis were included in the regression model for multivariate logistic regression analysis. The receiver operating characteristic (ROC) curve was calculated from the logistic regression model. The area under the curve (AUC) was assessed to evaluate the strength of prediction. *p*<0.05 was considered to be statistically significant.

## Results

### Clinical characteristics of asymptomatic and mild patients with COVID-19 on admission

Clinical data of a total of 991 patients, including asymptomatic (n = 705), and mild (n = 286) patients were collected. Table 1 showed the clinical characteristics of the asymptomatic and mild patients infected with COVID-19. There was no significant difference in age, gender, and underlying comorbidities in patients between the two groups. There was a significant difference between the asymptomatic group and the mild symptom group in the number of people who received the second dose of the vaccine (34.18% vs. 42.65%, p = 0.013).

**Table 1:** Clinical characteristics of asymptomatic,mild and severe patients with COVID-19 on admission

	Asymptomatic N = 705	Mild N = 286	Severe N = 166	*P* Value^*^Asymptomatic versus mild	*P* Value^*^mild versus severe	Overall N = 1157(% or IQR)
Clinical characteristics						
Sex				0.292	0.736	
Male	365(51.77%)	159(55.59%)	95(57.22%)			619(53.50)
Female	340(48.22%)	127(44.40%)	71(42.77%)			538(46.49)
Age	30(21.25, 46.00)	33(24,46)	62(44.50,72.50)	0.783	< 0.001	
Disease onset time (day)	1.00(1.00,1.00)	1.00(1.00,1.00)	1.50(1.00,6.00)	0.753	< 0.001	
COVID-19 vaccine						
0-dose	121(17.16%)	43(15.03%)	153(92.16%)	0.451	< 0.001	317(27.39)
1-dose	15(2.12%)	7(2.44%)	1(0.60%)	0.813	0.194	23(1.98)
2-doses	241(34.18%)	122(42.65%)	6(3.61%)	0.013	< 0.001	369(31.89)
3-doses	328(46.52%)	114(39.86%)	6(3.61%)	0.057	< 0.001	448(38.72)
Time from last vaccine dose (day)	83(49.00,161.00)	130(56.00,175.00)	86(55.00,153.5.00)	0.06	0.06	
Comorbidities						
Hypertension	64(9.07%)	23(8.04%)	87(52.40%)	0.624	< 0.001	174(15.03)
Coronary artery disease	18(2.55%)	14(4.89%)	40(24.09%)	0.073	< 0.001	72(6.22)
Diabetes	30(4.25%)	10(3.49%)	44(26.50%)	0.602	< 0.001	84(7.26)
COPD	2(0.28%)	3(1.04%)	14(8.43%)	0.148	0.012	19(1.64)
Cerebrovascular disease	11(1.56%)	3(1.04%)	43(25.90%)	0.58	< 0.001	57(4.92)
Chronic renal disease	7(0.99%)	2(0.69%)	14(8.43%)	0.737	< 0.001	23(1.98)
Malignant tumor	9(1.27%)	4(1.39%)	19(11.44%)	1	0.004	32(2.76)
Rheumatic disease	4(0.56%)	1(0.34%)	2(1.20%)	1	0.39	7(0.60)
Symptoms						
Fever		132(46.20%)	48(28.91%)		0.005	180(15.55)
Cough		168(58.70%)	62(37.34%)		0.002	230(19.87)
Shortness of breath		0	12(7.22%)		< 0.001	12(1.03)
Fatigue		29(10.10%)	12(7.22%)		0.299	41(3.54)
Nausea or vomiting		2(0.70%)	6(3.61%)		0.023	8(0.69)
Diarrhea		3(1.00%)	2(1.20%)		0.882	5(0.43)
Sore throat		91(31.80%)	5(3.01%)		< 0.001	96(8.29)
Laboratory findings						
WBC (10^9/L)	4.80(3.92,6.31)	4.68(4.06,6.85)	6.71(4.72,9.60)	0.582	< 0.001	5.39 (4.23,7.59)
EOS (10^9/L)	0.06(0.02,0.13)	0.05(0.01,0.09)	0.01(0.00,0.03)	0.065	< 0.001	0.04 (0.01,0.08)
Neutrophils (10^9/L)	2.59(1.94, 3.66)	2.87(1.97,4.31)	4.74(3.03,7.76)	0.873	< 0.001	3.40 (2.31,5.24)
Lymphocytes (10^9/L)	1.47(1.00,1.47)	1.56(0.75,2.14)	0.84(0.53,1.29)	0.319	< 0.001	1.29 (0.76,1.63)
Monocytes (10^9/L)	0.51(0.39,0.66)	0.55(0.39, 0.7)	0.50(0.35,0.75)	0.042	0.772	0.52 (0.38,0.70)
CRP (mg/L)	1.96(0.40,3.92)	2.68(0.90,3.25)	30.85(6.21,76.53)	0.782	< 0.001	14.92(2.50,27.90)
D-Dimer(ug/ml)	0.23(0.18, 0.34)	0.49(0.30,0.71)	1.42(0.93,2.84)	0.096	< 0.001	0.71 (0.47,1.30)
NLR	1.82(1.18, 2.84)	2.16(1.24, 3.65)	5.23 (2.74,11.92)	0.231	< 0.001	3.07 (1.72,6.14)
CD4 (cell/ul)		720.50(553.59, 943.25)	292.52(178.60,482.76)		< 0.001	366.09(366.09,713.01)
CD8 (cell/ul)		458.50(360.75, 609.00)	156.00(87.88,342.32)		< 0.001	307.25(224.32,475.66)
CD4/CD8		720.50(553.59, 943.25)	292.52(178.60,482.76)		0.074	506.51(366.10,713.01)
Outcome						
HFNO	0	0	40(24.10%)			40(3.45)
Mechanical ventilation	0	0	126(75.90%)			126(10.89)
Death	0	0	69(41.57%)			69(5.96)

Data are presented as median (IQR) or n (%). p values comparing the group of asymptomatic and mild patients are from χ² test or Mann-Whitney U test. EOS, Eosinophils. NLR, Neutrophil-to-Lymphocyte Ratio

For blood tests, EOS counts between the mild group and the asymptomatic group showed no significant difference (0.05 vs. 0.06, p = 0.065). There was no difference in the neutrophils counts (p = 0.873) and lymphocytes counts (p = 0.319), but Monocytes (p = 0.042) were found to be increased in the mild group compared with the asymptomatic group. (Table 1)

### Clinical characteristics of mild and severe patients with COVID-19 on admission

There was no significant difference in gender (p = 0.292), but a significant difference in age between the mild and severe patients. The median age of the mild and severe patients was 35.0 years (IQR 25–48) vs. 62 years (IQR 44.5–72.5), with a significant difference (p < 0.001). Significant differences in the underlying comorbidity include hypertension (p < 0.001), Coronary artery disease (p < 0.001), Diabetes mellitus (p < 0.001), Chronic renal disease(p < 0.001), and Cerebrovascular disease (p < 0.001) were identified between the mild and severe group of patients. **(**Table 1)

The most common symptoms including fever (p = 0.005), cough (p = 0.002), nausea or vomiting (p = 0.023), sore throat (p < 0.001), and shortness of breath (p < 0.001) were found between the mild and severe groups of patients. The median interval from the onset of symptoms to hospital admission for the mild and severe patients were 1.08 and 4.418 days, with a significant difference between the two groups (p < 0.001). Out of the 166 severe patients, 40 severe patients required high-flow nasal oxygen (HFNO), 126 severe patients required intubation followed by mechanical ventilation, and 69 out of 166 severe patients died finally. **(**Table 1)

For blood parameters, there was a significant difference in the median counts of lymphocytes (1.55 vs. 0.84, p < 0.001), WBC (5.35 vs. 6.71, p < 0.001), EOS (0.065 vs. 0.01, p < 0.001), and the significant difference of neutrophils (2.84 vs. 4.74, p < 0.001) were also found between the mild and severe group. Declined median CD4 (720.5 vs. 292.52, p < 0.001) and CD8 T cell counts (458.5 vs. 156, p < 0.001), while increased CRP (0.4 vs. 30.85, p < 0.001) and D-dimer (0.25 vs. 1.42, p < 0.001) were identified in the mild and severe groups. (Table 1)

### Effects of age and the underlying comorbidities on circulating EOS

**Fig. 2:**
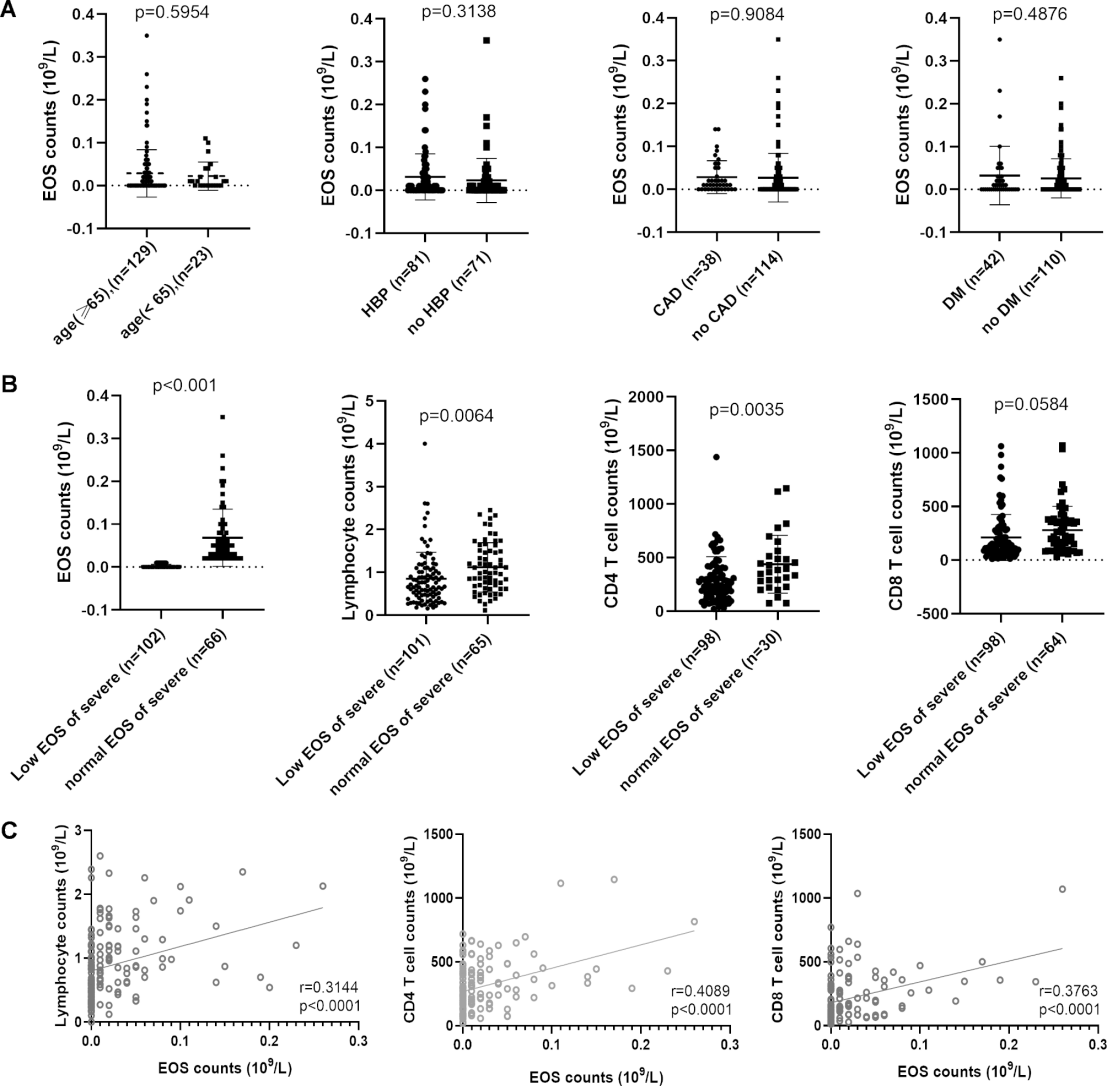
Effects of age and the underlying comorbidities on circulating EOS, and the correlation between circulating EOS and the CD4 or CD8 T cells HBP, high blood pressure; CAD, Coronary artery disease; DM, Diabetes mellitus

Table 1 showed that age and the underlying comorbidities were associated with the disease severity. Therefore, we furthermore evaluated the potential effect of age and the underlying comorbidities on circulating EOS. Our results showed that age (p = 0.595) and underlying comorbidities including HBP (p = 0.314), CAD(p = 0.908), and Diabetes(p = 0.488) had no significant effects on circulating EOS in the severe patients (Fig. 2A). This result suggests that age and underlying comorbidities have no significant effects on circulating EOS, which indicates that EOS likely is a predictor of disease severity independent of age and the underlying comorbidities.

### Correlation between circulating EOS counts and the T cell immunity in the mild and severe patients

Our results showed that circulating EOS, lymphocytes, CD4, and CD8 T cells were significantly decreased, while CRP and NLR significantly increased in the severe patients, compared with the mild patients (Table 1). In order to evaluate the correlation between circulating EOS and T cell immunity, we divided patients into two groups, including the low EOS group (< 0.02 × 10^9^/L) and the normal EOS group (≥ 0.02 × 10^9^/L) according to circulating EOS when admitted to the hospital.

In the mild patients, the median cell counts of CD4 (595 vs. 739, p = 0.069), CD8 T cells (359 vs. 486, p = 0.182), and Lymphocytes (1.18 vs. 1.7, p < 0.001), along with the median CRP (1.99 vs. 0.4, p < 0.001) and NLR (2.57 vs. 1.97, p = 0.075), were identified between the low EOS group (< 0.02 × 10^9^/L) and normal EOS Group (≥ 0.02 × 10^9^/L) (Table 2). While, in the severe patients, the median cell counts of CD4 (272.23 vs. 353.49, p = 0.011), CD8 (140 vs. 219.75, p = 0.058), and Lymphocytes (0.7 vs. 0.99, p = 0.006), along with median CRP (41.49 vs. 24.72, p = 0.001) and NLR (7.79 vs. 3.85, p = 0.026), were demonstrated between the low EOS group (< 0.02 × 10^9^/L) and normal EOS Group (≥ 0.02 × 10^9^/L) (Table 3; Fig. 2B).

**Table 2:** Correlation between circulating EOS and the T cell immunity in the mild patients

Mild patients	Low EOS (< 0.02 × 10^9/L) (n = 19)	Normal EOS (≥ 0.02 × 10^9/L) (n = 131)	P value
Sex			0.466
Male	10 (52.63%)	81 (61.83%)	
Female	9 (47.37%)	50 (38.17%)	
Age	36(31,52)	37(26,49)	0.693
WBC (10^9/L)	5.15(3.90,5.89)	5.9(4.45,6.95)	0.002
EOS (10^9/L)	0.01(0.01,0.01)	0.08(0.04,0.15)	< 0.001
Neutrophils (10^9/L)	3.34(2.48,4.07)	3.25(2.51,4.475)	0.129
Lymphocytes (10^9/L)	1.18(1.07,1.40)	1.7(1.45,2.045)	< 0.001
Monocytes (10^9/L)	0.44(0.36,0.60)	0.45(0.40,0.59)	0.376
CRP(mg/L)	1.99(0.48,11.55)	0.4(0.40,1.20)	< 0.001
D-Dimer(ug/ml)	0.14(0.14,0.14)	0.25(0.165,0.360)	0.805
CD4 (cell/ul)	595(468.00,830.00)	739 (559.18,898.50)	0.065
CD8 (cell/ul)	359(273.00,508.00)	486(373.50,618.00)	0.182
CD4/CD8	1.72(1.27, 1.94)	1.57(1.20, 2.11)	0.871
NLR	2.57(1.72, 3.80)	1.97(1.60, 2.54)	0.075

Data are presented as median (IQR) or n (%). p values comparing the group of mild and severe patients are from χ² test or Mann-Whitney U test. EOS, Eosinophils. NLR, Neutrophil-to-Lymphocyte Ratio

**Table 3:** Correlation between circulating EOS and the T cell immunity in the severe patients

Severe patients	Low EOS (< 0.02 × 10^9/L) (n = 101)	Normal EOS (≥ 0.02 × 10^9/L) (n = 65)	P value
Sex			0.307
Male	61(60.40%)	34 (52.31%)	
Female	40 (39.60%)	31(47.69%)	
Age	78(78.00,92.50)	81(74.00,90.00)	0.195
WBC (10^9/L)	7.05(5.32,10.18)	6.64(4.26,8.88)	0.102
EOS (10^9/L)	0(0.00,0.00)	0.04(0.02,0.08)	< 0.001
Neutrophils (10^9/L)	5.44(3.95, 8.54)	4.48(2.40,7.12)	0.026
Lymphocytes (10^9/L)	0.7(0.46,1.03)	0.99(0.70,1.50)	0.006
Monocytes (10^9/L)	0.465(0.31,0.77)	0.5(0.38,0.71)	0.825
CRP(mg/L)	41.96(10.89,96.27)	24.72(3.97,63.33)	0.001
D-Dimer(ug/ml)	1.42(0.96,3.55)	1.41(0.79,2.74)	0.365
CD4 (cell/ul)	272.23(138.23,407.49)	353.49(220.85,487.25)	0.011
CD8 (cell/ul)	140(81.02,291.47)	219.75(103.57,368.08)	0.058
CD4/CD8	1.7(0.9,2.59)	1.31(1.01,2.02)	0.229
NLR	7.79(3.92, 15.45)	3.85(2.34, 8.01)	0.026

Data are presented as median (IQR) or n (%). p values comparing the group of mild and severe patients are from χ² test or Mann-Whitney U test. EOS, Eosinophils. NLR, Neutrophil-to-Lymphocyte Ratio

### Effects of inactivated COVID-19 vaccine on circulating EOS

Next, we compared the effect of inactivated COVID-19 vaccine on circulating EOS and other factors associated with the activated inflammatory response such as CRP and NLR, according to the vaccination doses, and the time from the last anti-COVID-19 vaccine dose to the symptom onset. Because of the extremely low vaccination rate (13 out of 166 patients, 7.83%) in severe patients in this study, we only evaluated the effect of the inactivated COVID-19 vaccine on circulating EOS, both in the asymptomatic and the mild groups.

**Fig. 3:**
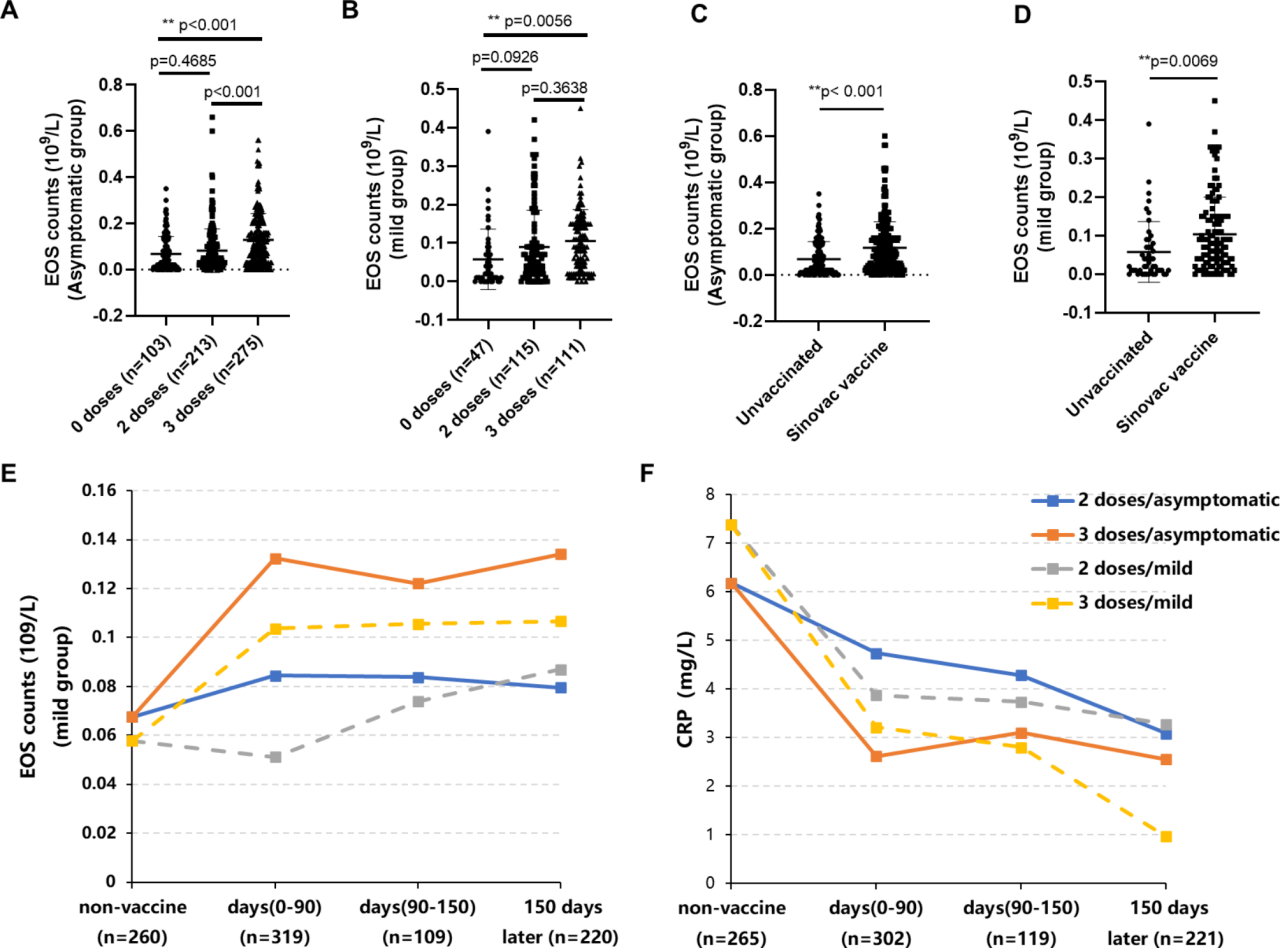
Effects of the inactivated COVID-19 vaccine on circulating EOS. The promoting effect of 2 doses or 3 doses of COVID-19 vaccine on circulating EOS in the asymptomatic (Fig. 3A, B and E); The effect of COVID-19 vaccines from different manufacturers (Fig. 3C and D); The role of COVID-19 vaccine on CRP levels (Fig. 3F). Non-vaccine: Not vaccinated; Days (0–90), Days (90–150), 150 days later: Time from the last anti-COVID-19 vaccine dose to the onset of symptoms

Our results showed that both 2 doses and 3 doses of the inactivated COVID-19 vaccine can promote circulating EOS in asymptomatic or mild patients. In particular, the 3rd booster shot of the COVID-19 vaccine demonstrated a more effective and sustained promoting effect on circulating EOS both in the asymptomatic (Fig. 3A) and mild patients (Fig. 3B). Interestingly, this promoting effect lasted more than five months later after the last anti-COVID-19 vaccine dose (It has not been evaluated for a longer time), both in the asymptomatic and the mild (Fig. 3E) groups. Furthermore, our results showed that both 2 doses and 3 doses of the COVID-19 vaccine could inhibit CRP levels in the asymptomatic and the mild groups. Inconsistent with circulating EOS, the 3rd booster shot of COVID-19 vaccine demonstrated a more significant inhibitory effect on CRP. (Fig. 3F)

Moreover, we also compared the effect of COVID-19 vaccines on circulating EOS. Our results show the inactivated COVID-19 vaccine (Sinovac, China) is likely to have a significant promoting effect on EOS, both in the asymptomatic (Fig. 3C) and the mild (Fig. 3D) patients.

### Predictive values of circulating EOS for disease severity

In this study, univariate analysis showed that there was a significant difference in age, underlying comorbidities, EOS, lymphocytes, CRP, CD4, and CD8 T cells between the mild and severe patients. Multi-factor logistic regression analysis was performed. Our results showed that EOS (OR 1.34, p = 0.006), CD8 T cell (OR 0.999, p = 0.024) and CD4 T cell (OR 0.995, p = 0.016) were independent risk factors for disease severity in patients with SARS-CoV-2 Omicron BA.2 variant infection. (Table 4)

**Table 4:** Multivariate logistic regression analysis in the mild and severe patients

Influencing factors	OR	95%CI	P value
age	1.15	1.08–1.21	< 0.001
HBP	1.68	0.38–7.38	0.491
CAD	1.06	0.16–6.99	0.945
DM	1.14	0.19–6.83	0.884
COPD	1.86	0.12–2.69	0.648
Chronic renal disease	1.15	0.19–6.85	0.798
WBC (10^9/L)	8.46	0.19–4.82	0.299
EOS (10^9/L)	1.34	1.24–1.42	0.006
Neutrophils (10^9/L)	0.11	0.00-7.21	0.308
Lymphocytes (10^9/L)	0.10	0.00-5.82	0.271
CRP(mg/L)	1.05	1.02–1.08	0.001
CD4 (cell/ul)	0.99	0.69–0.99	0.016
CD8 (cell/ul)	0.99	1.01–1.32	0.024

OR: odds ratio; CI: confidence interval

HBP, high blood pressure; CAD, Coronary artery disease; DM, Diabetes mellitus; COPD, Chronic Obstructive Pulmonary Disease. EOS, Eosinophils

**Fig. 4:**
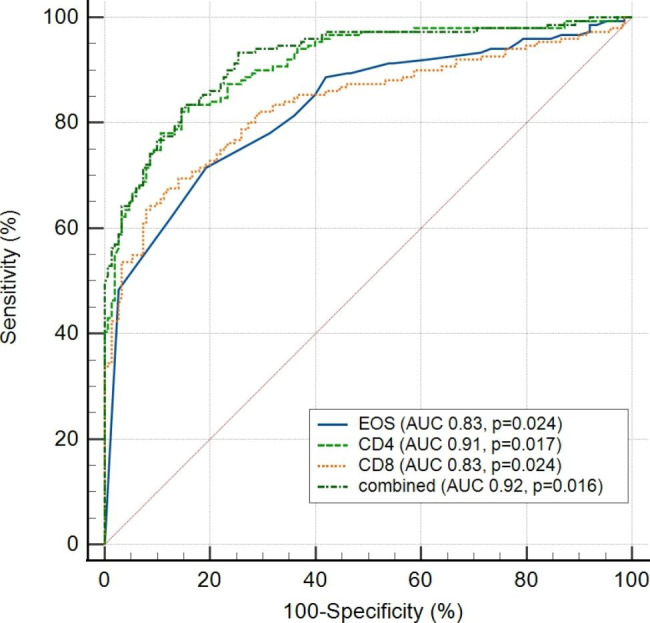
ROC curve analysis of circulating EOS for the disease severity EOS, Eosinophils; ROC: Receiver Operating Characteristics; Combined: combination of EOS and CD4 T cell

ROC curve analysis was conducted to calculate the area under the curve (AUC) of circulating EOS, CD4, and CD8 T cells between the mild and severe groups of patients. Our results showed the predictive value of EOS (AUC = 0.829, p = 0.0237), CD4 T cells (AUC = 0.910, p = 0.0167), or CD8 T cells (AUC = 0.834, p = 0240) for the disease severity. Moreover, the combination of the EOS and CD4 (AUC = 0.919, p = 0.0157) indicated a better predictive value for disease severity than each indicator (Fig. 4; Table 5).

**Table 5:** ROC curve analysis of EOS for disease severity

Variable	AUC	95% CI	P value
EOS	0.83	0.78 to 0.87	0.0237
CD4	0.91	0.87 to 0.93	0.0167
CD8	0.83	0.78 to 0.87	0.0240
Combined*	0.92	0.882to 0.94	0.0157

*Combined: Combination of EOS and CD4.

EOS, Eosinophils. ROC, Receiver operating characteristics

## Discussion

In addition to chest imaging findings, disease severity and laboratory findings should be taken into consideration for the determination of risk factors for COVID-19 patients. Studies of Epidemiological statistics have shown that “age over 65” was associated with higher risk of mortality [20, 21]. In this study, the mean age of the severe patients was 78.1 years old, which was significantly higher than that in the mild group (38.9 years old). This result indicated that old age still is a significant risk factor associated with the disease severity of the patients infected with SARS-CoV-2 Omicron/BA.2, which is consistent with previous studies on COVID-19 [22, 23]. Among the asymptomatic patients, 121 (17.16%) patients did not receive vaccine, 15 (2.12%) patients received one dose, 241 (34.18%) patients received two doses, and 328 (46.52%) patients received three doses of vaccine. No significant difference in age and the underlying comorbidities including HBP, CAD, DM, COPD, and Cerebrovascular disease of each group of patients was shown between the asymptomatic and the mild groups. While there was a significant difference in the underlying comorbidity between the mild and severe groups of patients. This result indicates that underlying comorbidities are significant risk factors for the disease severity prediction in these SARS-CoV-2 Omicron/BA.2 infected patients. The possible reason is that these underlying comorbidities may induce more serious inflammatory reactions [24, 25], resulting in severe and critical COVID-19, eventually contributing to death.

Our results showed that eosinopenia was common in the SARS-CoV-2 Omicron/BA.2 infected patients, which was consistent with previous studies on COVID-19 patients [26]. Our results showed that significantly decreased EOS was identified in the mild patients than in the asymptomatic patients. Notably, significantly decreased EOS was more common in severe patients. In this study, univariate analysis showed that the median circulating EOS was significantly different between the mild and severe groups (0.008 vs. 0.001, p < 0.001) (Table 1**)**. Multi-factor logistic regression analysis was performed and the result showed that EOS (OR 0.00, 95% CI 0.00-0.012, p = 0.008) likely is an independent risk factor for the disease severity prediction **(**Table 4**).** ROC curves analysis showed that EOS (AUC = 0.828, p = 0.024), the combination of EOS, and CD4 (AUC = 0.92, p = 0.016) demonstrated the predictive value for disease severity prediction (Fig. 3; Table 5).

Our results suggested that the underlying comorbidities may contribute to the disease severity of COVID-19 patients. A study has suggested that age and underlying comorbidities such as diabetes may be associated with dismal outcomes [27]. These risk factors may contribute to declined EOS or impaired T cell immunity. Thus, we further evaluated the potential effect of age and the underlying comorbidities on circulating EOS. However, our results demonstrated that no significant differences in EOS associated with age (p = 0.5954), HBP (p = 0.3138), CAD (p = 0.9084), or DM (p = 0.4876) in severe patients. Thus, circulating EOS likely is a predictor of severe disease independent of age and the underlying comorbidities.

Studies have indicated that immune injury is a risk factor associated with disease severity in COVID-19 patients [28–30]. Immune function injury is directly or indirectly associated with SARS-CoV-2 infection. However, the inflammatory factor is possibly due to the direct effect of the virus or the indirect effect of immune dysfunction. Our study showed that severe patients exhibited impaired T cell immunity and more activated inflammatory response, as demonstrated by the significantly decreased CD4 and CD8 T cell counts, while increased CRP levels or NLR ratio in the severe patients than that in mild patients. Therefore, we further evaluated the correlation between circulating EOS and CD4 or CD8 T cells both in mild and severe patients.

We divided COVID-19 severe patients into two groups based on the circulating EOS counts when admitted to the hospital, including the low EOS group (< 0.02 × 10^9^/L) and the normal EOS group (≥ 0.02 × 10^9^/L). Our results showed that patients with low EOS exhibited impaired T cell immune function and activated inflammatory response, as demonstrated by the significantly decreased circulating CD4, and CD8 T cells, while increased CRP level or NLR ratio, compared with the normal EOS group, both in the mild and severe patients (Fig. 2). Therefore, eosinophilia, along with low CD4, CD8 T cells, and activated inflammatory response, are likely to be significant prognostic factors for the disease severity.

We are still unable to conclude whether the decreased EOS or absence of EOS is due to SARS-CoV-2 infection. Therefore, the association between EOS and other immune factors should be characterized in future studies that involve larger cohorts of COVID-19 patients with different illness severities to determine whether they could be well used to predict disease outcomes.

We compared the effects of inactivated COVID-19 vaccine on circulating EOS. Our results showed both 2 doses and 3 doses of COVID-19 vaccine could promote circulating EOS. In particular, the 3rd booster shot of COVID-19 vaccine demonstrated a more effective and sustained promoting effect on EOS, while inhibiting the CRP level, both in the asymptomatic and mild patients (Fig. 3). Moreover, the longitudinal EOS data showed that the promoting effect of COVID-19 vaccine could last five months later after the last vaccination (It has not been evaluated for a longer time).

In this study, the vaccination rate was only 7.83% (13 out 166 severe cases) in severe patients. Studies have shown that the COVID-19 vaccine especially the 3-doses vaccine had protective effects against the SARS-CoV-2 variant, reducing the occurrence of disease severity and death [31–33]. Consistently, 3-doses of COVID-19 vaccine demonstrated a more effective and sustained promoting effect on circulating EOS, along with the attenuated inflammatory response (CRP).

SARS-COV-2 Omicron variants have emerged immune-escape to the existing COVID-19 vaccine [17, 34, 35]. Thus, besides the specific immune response of COVID-19 vaccine against SARS-COV-2, whether circulating EOS involved in the anti-COVID-19 immunity? Or is circulating EOS just a predictive marker for the responsiveness to COVID-19 vaccine or disease severity? Such issues still need to be clarified in the future.

Our study has some limitations. The symptoms of some patients were not well documented, and some patients had inadequate laboratory results due to variations in the structure of the electronic databases.

## Conclusions

Our results suggest that decreased EOS, along with impaired T cell immunity, as evidenced by the eosinopenia may be an independent risk factor for the prediction of poor clinical outcomes in patients with SARS-CoV-2 Omicron/BA.2 variant infection. Both 2-doses and 3-doses of the COVID-19 vaccine can promote EOS. In particular, the 3rd booster shot of inactivated COVID-19 vaccine demonstrates a sustained promoting effect on circulating EOS. However, whether and how EOS participates in the antiviral effects of the COVID-19 vaccine remains to be elucidated.

## Data Availability

The datasets used and analyzed during the current study available from the corresponding author on reasonable request.

## Abbreviations

EOS: Eosinophils
COVID-19: 2019 coronavirus disease
SARS-CoV-2: Severe acute respiratory syndrome coronavirus 2
HBP: High blood pressure
CAD: Coronary artery disease
DM: Diabetes mellitus
COPD: Chronic Obstructive Pulmonary Disease
HFNV: High-flow nasal oxygen
IMV: Invasive mechanical ventilation
ICU: intensive care unit
CRP: C-Reactive Protein
IQR: Interquartile range
NLR: Neutrophil-to-Lymphocyte Ratio
ROC: Receiver operating characteristics
WBC: White blood cell
WHO: World Health Organization
